# Supplementary material for: Inhibiting WNT and NOTCH in renal cancer stem cells and the implications for human patients
Source: Nat Commun. 2020 Feb 17;11:929. doi: 10.1038/s41467-020-14700-7 (PMC7026425; doi:10.1038/s41467-020-14700-7)
Supplement: Supplementary file 1 — Supplementary Information [file 41467_2020_14700_MOESM1_ESM.pdf]

Supplementary Information

Fendler et al.

Inhibiting WNT and NOTCH in renal cancer stem cells and the implications for human patients

Supplementary Figures

Supplementary Fig. 1. Analysis of ccRCC cancer stem cells using the stem cell markers CXCR4, MET, and CD44.____Page 3

Supplementary Fig. 2. Histological examination of xenografts and corresponding primary tumors.____Page 5

Supplementary Fig. 3. Analysis of ccRCC sphere and organoid cultures.________Page 7

Supplementary Fig. 4. Gene expression profiling in CXCR4+MET+CD44+, spheres and ccRCC tumors.____Page 9

Supplementary Fig. 5. Single-Cell Sequencing of CXCR4+MET+CD44+ cells.___Page 12

Supplementary Fig. 6. Pharmacological inhibition of WNT and NOTCH signaling in ccRCC. ____Page 14

Supplementary Fig. 7. Inhibition of WNT and NOTCH signaling in patient-derived ccRCC xenografts.____Page 17

Supplementary Figure 8. FACS gating strategy____________________________Page 19

Supplementary Tables

Supplementary Table 1. Clinical characteristics of ccRCC patients.____________Page 21

Supplementary Table 2. Clinical characteristics of ccRCC patients.____________Page 22

Supplementary Table 3. IC50-values of spheres treated with ICG-001 or DAPT__Page 23

Supplementary Table 4. Correlation of IC50 values with pathological stage, Fuhrman Grade or percentage of $CXCR4^{+}MET^{+}CD44^{+}$.___________________________________Page 24

Supplementary Table 5. RT-qPCR primer sequences.______________________Page 25

Supplementary Table 6. Antibodies_____________________________________Page 26

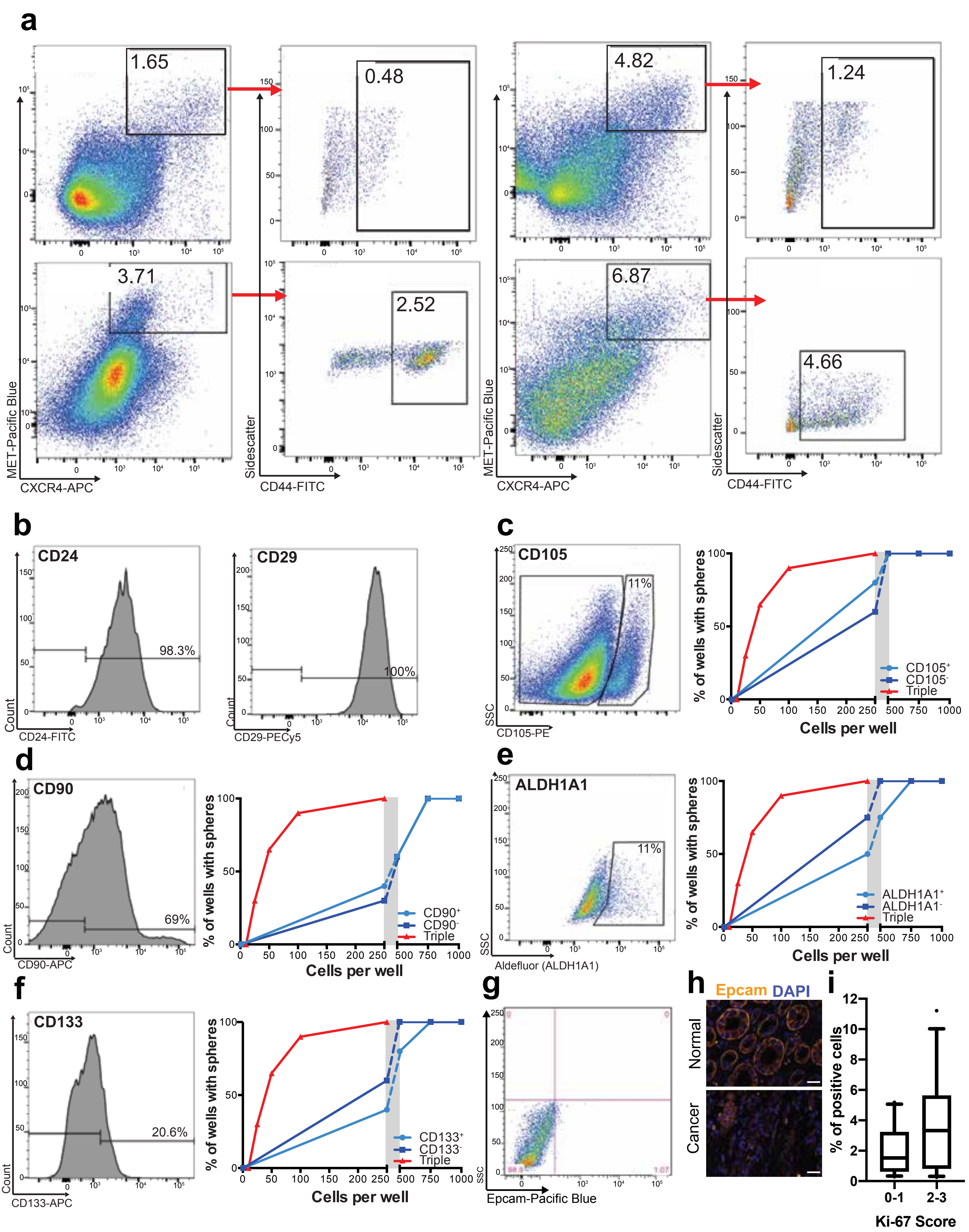
a
1.65
0.48
4.82
1.24
3.71
2.52
6.87
4.66
MET-Pacific Blue
CXCR4-APC
Sidescatter
CD44-FITC
b
CD24
98.3%
Count
CD24-FITC
CD29
100%
CD29-PECy5
c
CD105
11%
SSC
CD105-PE
% of wells with spheres
Cells per well
CD105+
CD105-
Triple
d
CD90
69%
CD90-APC
CD90+
CD90-
Triple
e
ALDH1A1
11%
Aldefluor (ALDH1A1)
ALDH1A1+
ALDH1A1-
f
CD133
20.6%
CD133-APC
CD133+
CD133-
g
Epcam-Pacific Blue
h
Epcam DAPI
Normal
Cancer
i
% of positive cells
0-1
2-3
Ki-67 Score

Supplementary Figure 1. Analysis of ccRCC cancer stem cells using the stem cell markers CXCR4, MET, and CD44.

(a) Representative FACS of single cell suspensions of ccRCC cells using the markers CXCR4, MET and CD44. (b-f) Representative FACS and accompanying limiting dilution assays using various other surface markers. Limiting dilution assays were not performed for CD24 and CD29-sorted cells. (g) Representative FACS using the epithelial marker Epcam. (h) Immunofluorescence for Epcam in ccRCC tissue and normal adjacent kidney (scale bars, 25 µm) Epcam staining was repeated in 5 independent ccRCC tumors. (i) Percentage of $CXCR4^{+}MET^{+}CD44^{+}$ in ccRCC with low (0-1, n=16) or high (2-3, n=21) Ki-67 scores. Ki-67 was scored from 0 (no positive nuclei) to 3 (all/ almost all nuclei positive). Boxes show the 25, 50 and 75 percentiles, whiskers the 10 and 90 percentiles, outliers are shown as dots.

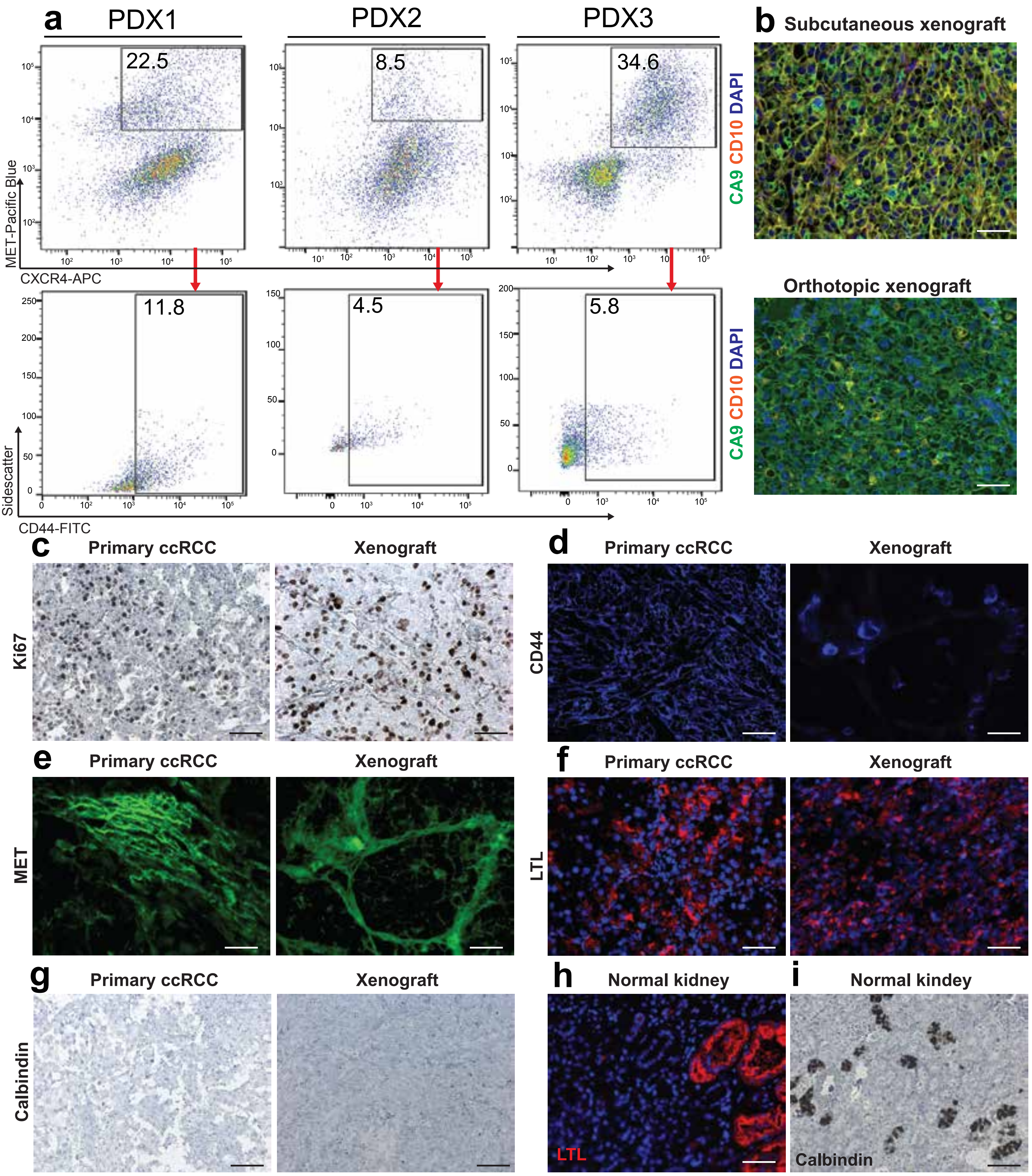
a
PDX1
PDX2
PDX3
22.5
8.5
34.6
MET-Pacific Blue
CXCR4-APC
11.8
4.5
5.8
Sidescatter
CD44-FITC
b
Subcutaneous xenograft
CA9 CD10 DAPI
Orthotopic xenograft
CA9 CD10 DAPI
c
Primary ccRCC
Xenograft
Ki67
d
Primary ccRCC
Xenograft
CD44
e
Primary ccRCC
Xenograft
MET
f
Primary ccRCC
Xenograft
LTL
g
Primary ccRCC
Xenograft
Calbindin
h
Normal kidney
LTL
i
Normal kindey
Calbindin

Supplementary Figure 2. Histological examination of xenografts and corresponding primary tumors

(a) Representative FACS of single cell suspensions of subcutaneous xenografts using the markers CXCR4, MET and CD44. Axis label of sidescatter is a multiple of 1000. (b) Representative immunohistochemistry of CA9 and CD10 in subcutaneous xenografts and a corresponding orthotopic xenograft (scale bars, 50 μm). (c) Representative immunohistochemistry of Ki-67 in ccRCC and a corresponding subcutaneous xenograft (scale bars, 100 μm). (d) Representative immunofluorescence of CD44 in different ccRCCs (left dedifferentiated solid histology, right nested clear cells) (scale bars, 50 μm). (e) Representative immunofluorescence of MET in different ccRCCs (left tumor border, right center) (scale bars, 50 μm). (f) Representative immunofluorescence of LTL in ccRCC and a corresponding subcutaneous xenograft (scale bars, 50 μm). (g) Representative immunohistochemistry of Calbindin in ccRCC and a corresponding subcutaneous xenograft (scale bars, 100 μm). (h) Representative stainings of LTL (scale bars, 50 μm) and Calbindin (scale bars, 100 μm) in normal adjacent kidney marking proximal tubules (LTL) or distal tubules (Calbindin). All stainings were performed in 3 independent xenografts and in 42 ccRCC specimens.

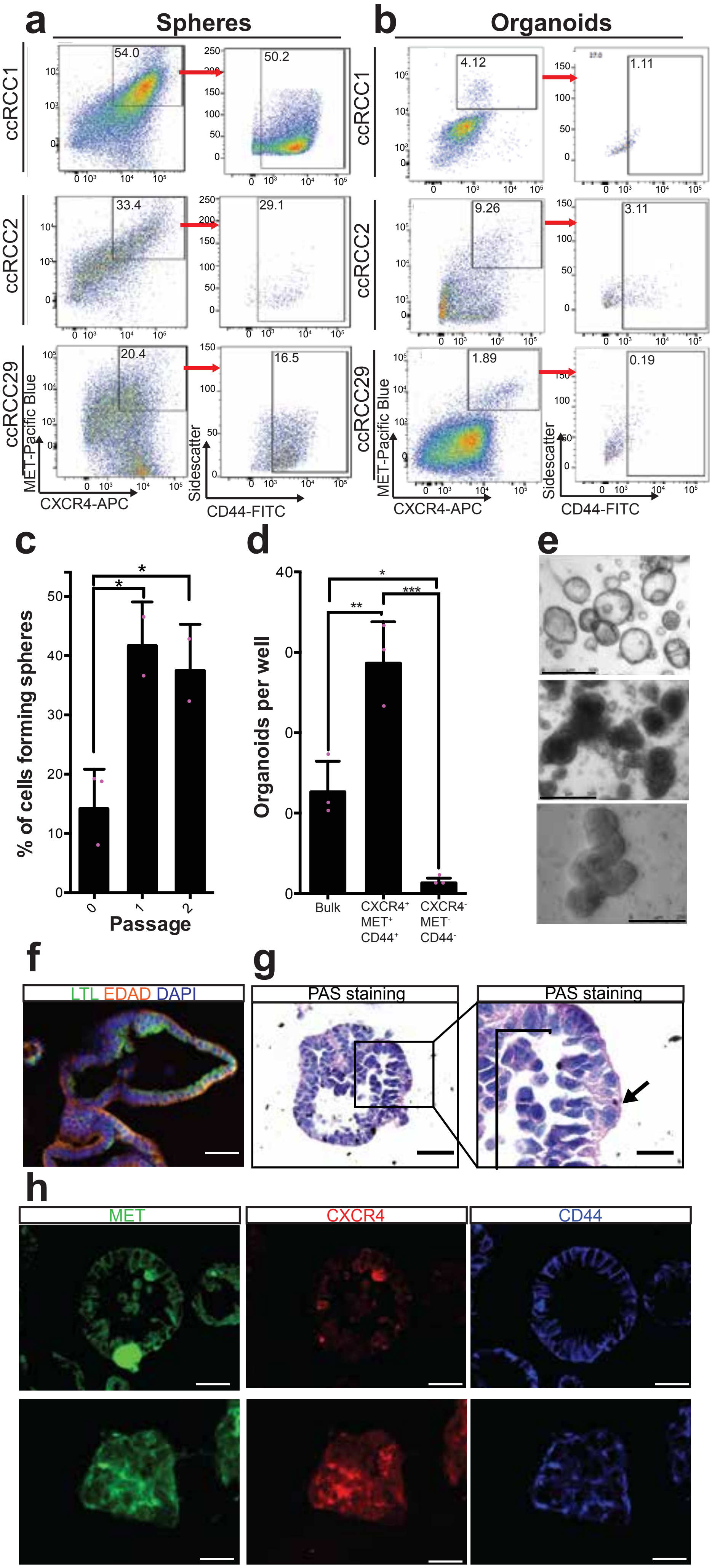
a
Spheres
b
Organoids
ccRCC1
ccRCC2
ccRCC29
MET-Pacific Blue
CXCR4-APC
Sidescatter
CD44-FITC
c
% of cells forming spheres
Passage
d
Organoids per well
Bulk
CXCR4+ MET+ CD44+
CXCR4- MET- CD44-
e
f
LTL EDAD DAPI
g
PAS staining
PAS staining
h
MET
CXCR4
CD44

Supplementary Figure 3. Analysis of ccRCC sphere and organoid cultures.

(a, b) Representative FACS images of single cell suspensions of 7 days cultured ccRCC spheres and organoids. Axis label of sidescatter is a multiple of 1000. (c) Sphere cultures were reseeded every 7 days and the numbers of spheres were counted for each passage (n=3 independent patients). Data are shown as mean, error bars represent standard deviation, p-values: * < 0.05 by one-way ANOVA with Dunnett's post test (two-sided). (d) Number of organoids formed after 7 days in culture from control ccRCC, $CXCR4^{+}MET^{+}CD44^{+}$ or $CXCR4^{-}MET^{-}CD44^{-}$ cells (n=3 independent patients). Data are shown as mean, error bars represent standard deviation, p-values: * < 0.05, ** < 0.01, *** < 0.001 by one-way ANOVA with Tukey post test (two-sided). (e) Representative brightfield images of organoids showing different morphologies, scale bars as indicated. Brightfield images were obtained from 15 independent organoid cultures. (f) Representative images of LTL localized to the apical side of an organoid (scale bars, 50 µm). LTL staining was performed in 10 independent organoid cultures. (g) Representative image of PAS staining for cytoplasmic glycogen, marked by an arrow (scale bars, 50 and 25 µm). (h) Representative images of MET, CXCR4 and CD44 in organoids (top) and spheres (bottom) (scale bars, 25 µm).

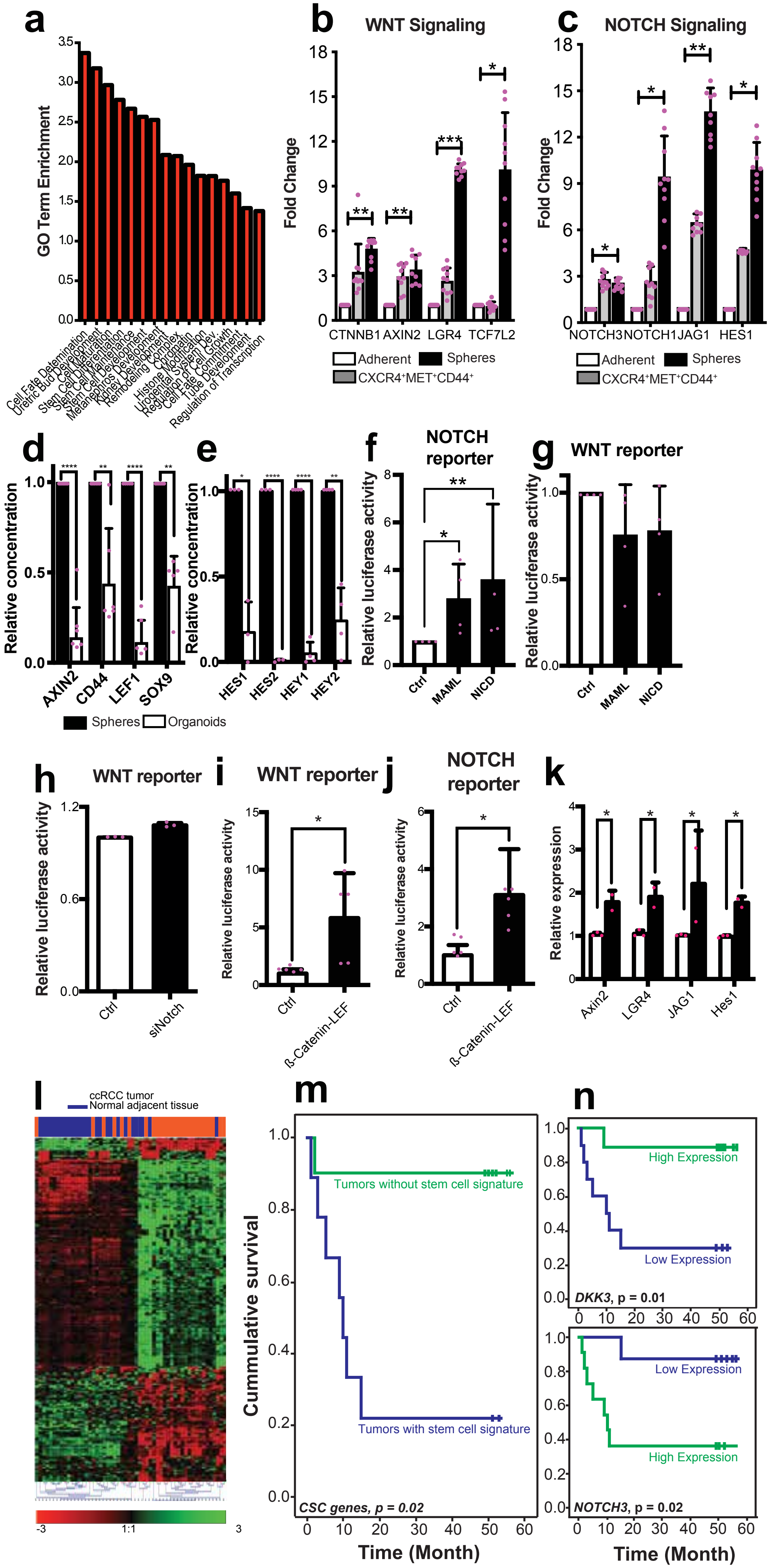

a
GO Term Enrichment
Cell Fate Determination
Ureteric Bud Development
Cell Maturation
Stem Cell Differentiation
Stem Cell Maintenance
Stem Cell Development
Metanephros Development
Kidney Development
Remodeling Complex
Histone Modification
Chromatin
Urogenital System Dev.
Regulation of Cell Growth
Cell Fate Commitment
Tube Development
Regulation of Transcription
b
WNT Signaling
Fold Change
CTNNB1 AXIN2 LGR4 TCF7L2
Adherent
Spheres
CXCR4+MET+CD44+
c
NOTCH Signaling
Fold Change
NOTCH3 NOTCH1 JAG1 HES1
Adherent
Spheres
CXCR4+MET+CD44+
d
Relative concentration
AXIN2 CD44 LEF1 SOX9
Spheres
Organoids
e
Relative concentration
HES1 HES2 HEY1 HEY2
f
NOTCH reporter
Relative luciferase activity
Ctrl MAML NICD
g
WNT reporter
Relative luciferase activity
Ctrl MAML NICD
h
WNT reporter
Relative luciferase activity
Ctrl siNotch
i
WNT reporter
Relative luciferase activity
Ctrl ß-Catenin-LEF
j
NOTCH reporter
Relative luciferase activity
Ctrl ß-Catenin-LEF
k
Relative expression
Axin2 LGR4 JAG1 Hes1
l
ccRCC tumor
Normal adjacent tissue
-3
1:1
3
m
Cummulative survival
Tumors without stem cell signature
Tumors with stem cell signature
CSC genes, p = 0.02
Time (Month)
n
High Expression
Low Expression
DKK3, p = 0.01
Low Expression
High Expression
NOTCH3, p = 0.02
Time (Month)

Supplementary Figure 4. Gene expression profiling in CXCR4+MET+CD44+, spheres and ccRCC tumors.

(a) GO term analysis of deregulated genes in $CXCR4^{+}MET^{+}CD44^{+}$ and sphere cells. (b, c) Expression of WNT and NOTCH pathway genes in control cells, $CXCR4^{+}MET^{+}CD44^{+}$ cells and spheres derived from ccRCC tumors (n=10). Data are shown as mean, error bars represent standard deviation. p-values: * < 0.05, ** < 0.01, *** < 0.001 by one-way ANOVA (two-sided). (d) Comparative expression analysis of WNT target genes in spheres and organoids (n = 5, matched samples from independent patients). Data are shown as mean, error bars represent standard deviation, p-values: ** < 0.01, **** < 0.0001 by two-sided t-test. (e) Comparative expression analysis of NOTCH target genes in spheres and organoids (n = 5, matched samples from independent patients). Data are shown as mean, error bars represent standard deviation, p-values: * < 0.05, ** < 0.01, **** < 0.0001 by two-sided t-test. (f) NOTCH luciferase reporter gene assay in sphere cultures transfected with either a MAML1 or NICD-expressing plasmid (n=4 independent patients). Data are shown as mean, error bars represent standard deviation. p-values: * < 0.05, ** < 0.01 by RM ANOVA with Dunnett's post test (two-sided). (g) WNT luciferase reporter gene assay in sphere cultures transfected with either a MAML1 or NICD-expressing plasmid (n=4 independent patients). Data are shown as mean, error bars represent standard deviation. (h) WNT luciferase reporter gene assay in sphere cultures transfected with siRNA against (n=3 independent patients). Data are shown as mean, error bars represent standard deviation. (i,j,k) WNT or NOTCH luciferase reporter gene assays of sphere cultures (n=5 independent patients) transfected with a plasmid controlling expression of a β-Catenin-LEF1 fusion protein. Data are shown as mean, error bars represent standard deviation, p-values: * < 0.05, by two-sided t-test. (l) Heatmap showing the differential expression of the stem cell signature in ccRCC specimens (orange, n=28) and normal adjacent tissue (blue, n=28). (m) Overall survival of ccRCC patients with or without the stem cell signature. Predictor score for each patient was calculated by Cox regression analysis. Patients were stratified into groups according to the median predictor score. (n) Overall survival of ccRCC patients with high (expression ≥ median) or low (expression <

median) expression of JAG1, NOTCH3, EP300 and DKK3. Censored patients are presented as vertical lines. Significance was tested by log rank test (two-sided).

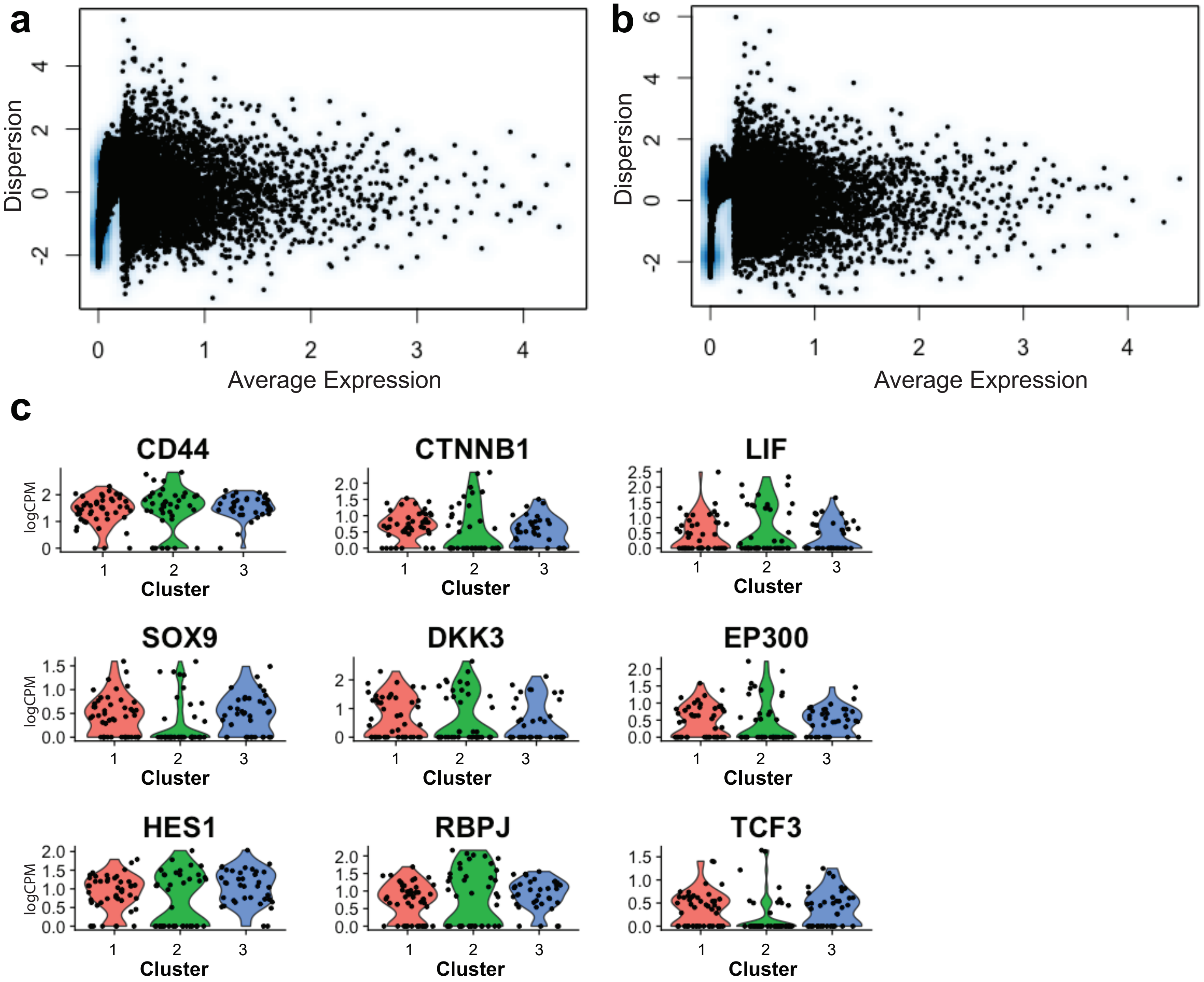
a
b
Dispersion
Average Expression
c
CD44
CTNNB1
LIF
SOX9
DKK3
EP300
HES1
RBPJ
TCF3
logCPM
Cluster

Supplementary Fig. 5. Single-Cell Sequencing of CXCR4+MET+CD44+ cells.

(a, b) Most variable genes in single cells for each patient. (c) Expression of non-variant WNT, NOTCH and stem cell genes in each cluster. Data are shown as scatter plot, violin plot represents data density.

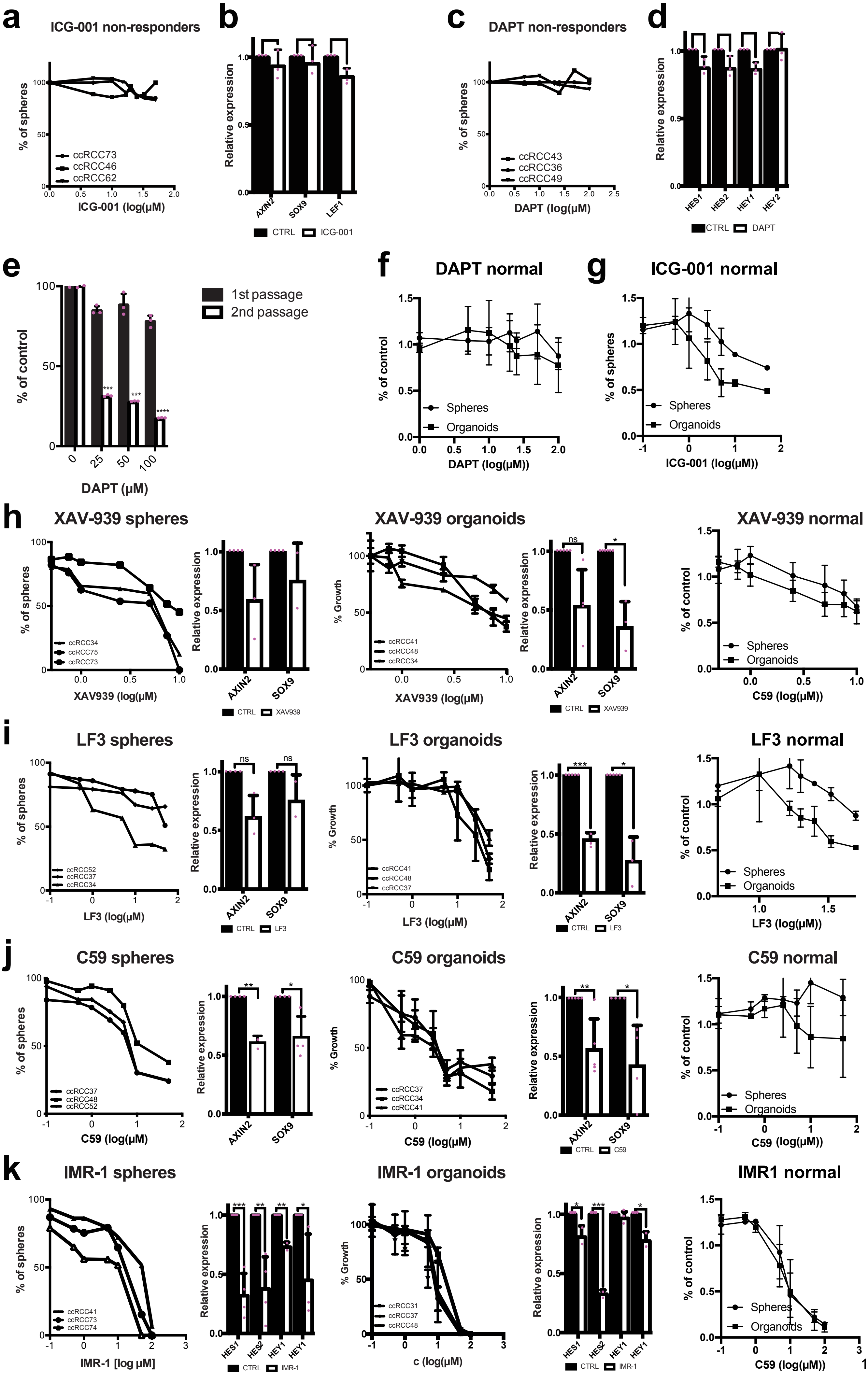
a
ICG-001 non-responders
% of spheres
ccRCC73
ccRCC46
ccRCC62
ICG-001 (log(μM))
b
Relative expression
AXIN2
SOX9
LEF1
CTRL
ICG-001
c
DAPT non-responders
% of spheres
ccRCC43
ccRCC36
ccRCC49
DAPT (log(μM))
d
Relative expression
HES1
HES2
HEY1
HEY2
CTRL
DAPT
e
% of control
1st passage
2nd passage
DAPT (μM)
f
DAPT normal
% of control
Spheres
Organoids
DAPT (log(μM))
g
ICG-001 normal
% of spheres
Spheres
Organoids
ICG-001 (log(μM))
h
XAV-939 spheres
% of spheres
ccRCC34
ccRCC75
ccRCC73
XAV939 (log(μM))
Relative expression
AXIN2
SOX9
CTRL
XAV939
XAV-939 organoids
% Growth
ccRCC41
ccRCC48
ccRCC34
XAV939 (log(μM))
XAV-939 normal
% of control
Spheres
Organoids
C59 (log(μM))
i
LF3 spheres
% of spheres
ccRCC52
ccRCC37
ccRCC34
LF3 (log(μM))
Relative expression
CTRL
LF3
LF3 organoids
% Growth
ccRCC41
ccRCC48
ccRCC37
LF3 (log(μM))
LF3 normal
% of control
Spheres
Organoids
LF3 (log(μM))
j
C59 spheres
% of spheres
ccRCC37
ccRCC48
ccRCC52
C59 (log(μM))
Relative expression
CTRL
C59
C59 organoids
% Growth
ccRCC37
ccRCC34
ccRCC41
C59 (log(μM))
C59 normal
% of control
Spheres
Organoids
C59 (log(μM))
k
IMR-1 spheres
% of spheres
ccRCC41
ccRCC73
ccRCC74
IMR-1 [log μM]
Relative expression
HES1
HES2
HEY1
HEY1
CTRL
IMR-1
IMR-1 organoids
% Growth
ccRCC31
ccRCC37
ccRCC48
c (log(μM))
IMR1 normal
% of control
Spheres
Organoids
C59 (log(μM))

Supplementary Figure 6. Pharmacological inhibition of WNT and NOTCH signaling in ccRCC.

(a) Representative inhibitor treatments of sphere cultures from 3 independent patients not responding to ICG-001. Sphere cultures were treated with 5 to 50 µM ICG-001 (b) Non-responders were treated with 20 µM ICG-001 (n=4 independent patients). Expression of target genes was measured by RT-qPCR. Data are shown as mean, error bars represent standard deviation. (c) Representative inhibitor treatments of sphere cultures from 3 independent patients not responding to DAPT. Sphere cultures were treated with 1 to 100 µM DAPT. (d) Non-responders were treated with 20 µM DAPT (n=4 independent patients). Expression of target genes was measured by RT-qPCR. Data are shown as mean, error bars represent standard deviation. (e) Organoids were treated with 0 to 100 µM DAPT for seven days. Cultures were reseeded and cultured with the same DAPT concentration for seven days (n=3 independent patients). Metabolic activity was measured by CellTiterGlo assay and normalized to vehicle treated controls. Data shown as mean, error bars represent standard deviation. (f,g) Normal kidney sphere cultures were treated with 5 to 50 µM ICG-001 or 1 to 100 µM DAPT for 7 days, and spheres < 25 µM were counted (n=5 independent patients). Data are shown as mean, error bars represent standard deviation. p-values: *** < 0.001, **** < 0.0001 by two-sided t-test.(h) Sphere and organoid cultures from ccRCC or normal kidney organoids (n=5 independent patients) were treated with 0.5 to 10 µM XAV-939, or treated with 7.5 µM XAV-939 (n=5 independent patients) for 24 hrs and the expression of target genes was measured by RT-qPCR. Data of ccRCC sphere and organoids are from single patients. Data of normal kidney organoids and RT-PCR data are shown as mean, error bars represent standard deviation. p-values: * < 0.05 by two-sided t-test. (i) Sphere and organoid cultures from ccRCC or normal kidney organoids (n=5 independent patients) were treated with 0.1 to 100 µM LF3, or treated with 25 µM LF3 (n=5 independent patients) for 24 hrs and the expression of target genes was measured by RT-qPCR. Data of ccRCC sphere and organoids are from single patients. Data of normal kidney organoids and RT-PCR data are shown as mean, error bars represent standard deviation. p-values: * < 0.05, *** < 0.0001 by two-sided t-test. (j) Sphere

and organoid cultures from ccRCC or normal kidney organoids (n=5 independent patients) were treated with 0.1 to 100 µM C59, or treated with 25 µM C59 (n=5 independent patients) for 24 hrs and the expression of target genes was measured by RT-qPCR. Data of ccRCC sphere and organoids are from single patients. Data of normal kidney organoids and RT-PCR data are shown as mean, error bars represent standard deviation. p-values: * < 0.05, ** < 0.001 by two-sided t-test. (k) Sphere and organoid cultures from ccRCC or normal kidney organoids (n=5 independent patients) were treated with 0.1 to 100 µM IMR-1, or treated with 15 µM IMR-1 (n=5 independent patients) for 24 hrs and the expression of target genes was measured by RT-qPCR. Data of ccRCC sphere and organoids are from single patients. Data of normal kidney organoids and RT-PCR data are shown as mean, error bars represent standard deviation. p-values: * < 0.05, ** < 0.001, *** < 0.001 by two-sided t-test.

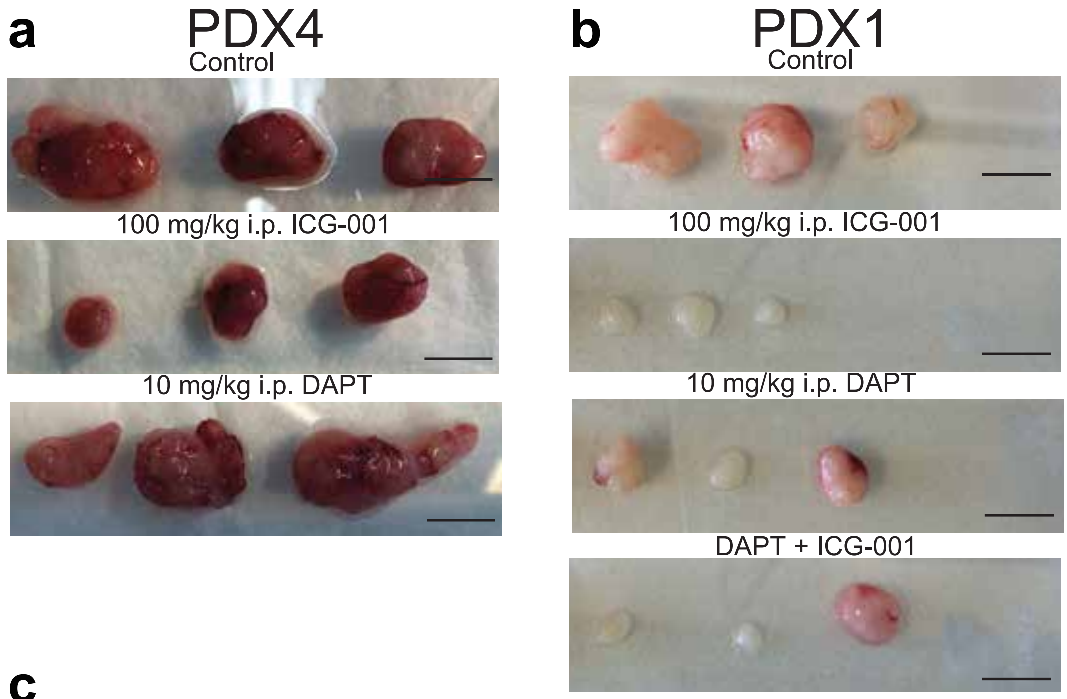

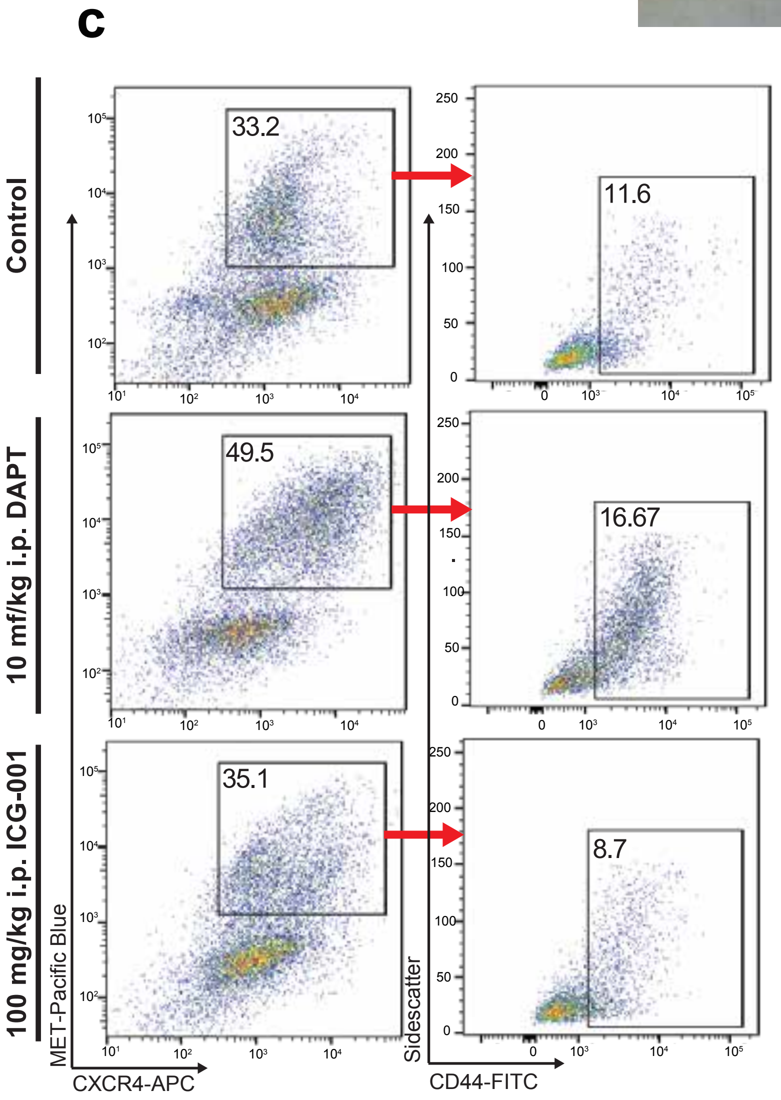

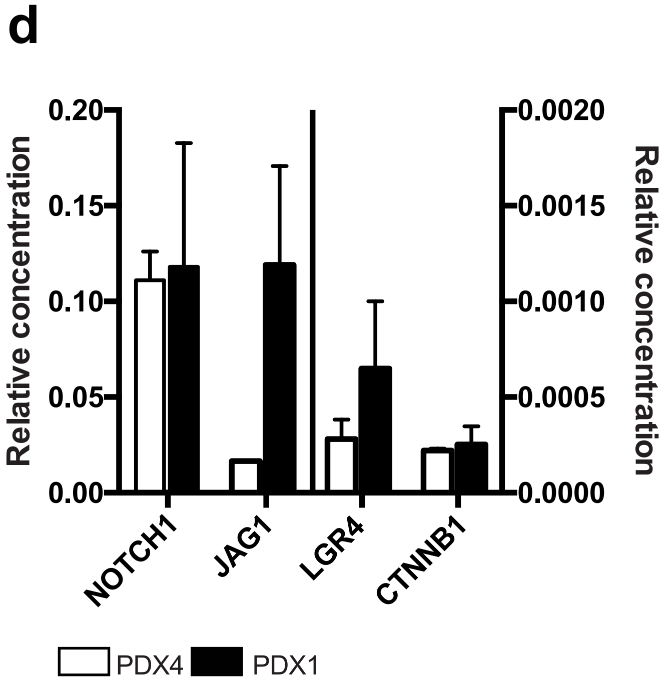

e

	ICG-001	DAPT
PDX4	+	-
ccRCC32	+	-
PDX1	+	+
ccRCC71	+	+

Supplementary Figure 7. Inhibition of WNT and NOTCH signaling in patient-derived ccRCC xenografts.

(a, b) Size of subcutaneous tumors after treatment with 100 mg/kg ICG-001, 10 mg/kg DAPT, combinations, or vehicle for 19 days (PDX4) or 23 days (PDX1) (scale bars, 1 cm). (c) FACS of subcutaneous tumors using the markers CXCR4, MET and CD44 after treatment with 100 mg/kg ICG-001, 10 mg/kg DAPT, combinations, or vehicle for 19 days (PDX4). Axis label of sidescatter is a multiple of 1000. (d) Comparative gene expression analysis of NOTCH1, JAG1 and CTNNB1 (left y-axis) and LGR4 (right y-axis) in PDX1 and PDX4 tumors without treatment. Data are shown as mean, error bars represent standard deviation from 3 technical replicates of each PDX. (e) Comparison of treatment response in CSC and xenografts from the same ccRCC tumor.

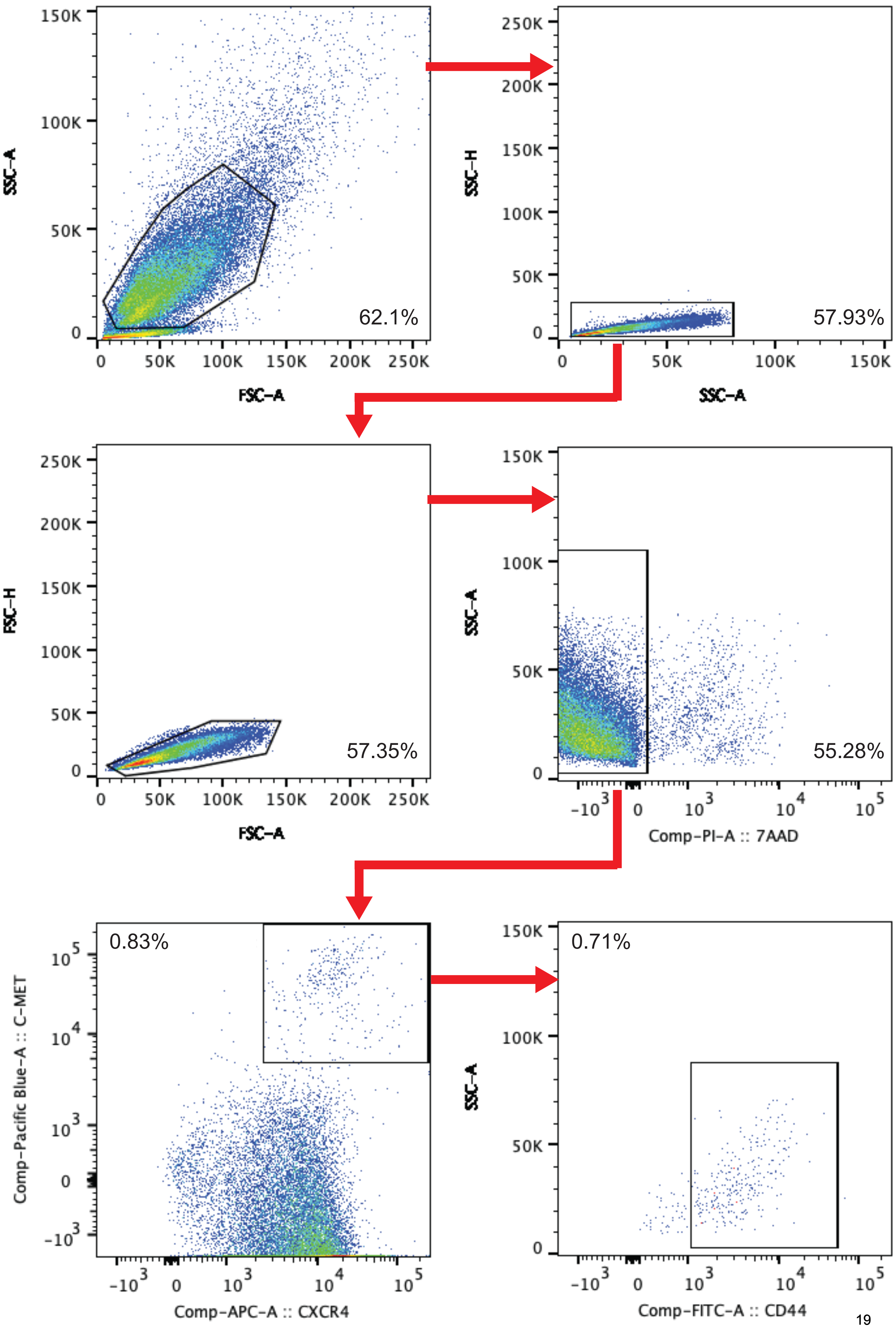

62.1%
57.93%
57.35%
55.28%
0.83%
0.71%
SSC-A
SSC-H
FSC-A
FSC-H
Comp-PI-A :: 7AAD
Comp-Pacific Blue-A :: C-MET
Comp-APC-A :: CXCR4
Comp-FITC-A :: CD44

Supplementary Figure 8. FACS gating strategy

Single cells were gated by plotting SSC versus FSC and confirmed by gating FSC-A vs FSC-H and SSC-A vs. SSC-H. Viable cells were identified by negative 7AAD staining. CXCR4^{+}MET^{+} cells were identified the viable cell population and further split by CD44. SSC, sidescatter, FSC, forwardscatter, A, Area, H, Height.

Supplementary Table 1: Clinical characteristics of ccRCC patients.

	Primary cells	
**Characteristics**	**Patients (n=55)**	**%**
**Age, years**		
Median	63	
Range	34-85	
**Sex**		
Male	35	64
Female	20	36
**Pathological stage**		
pT1	16	29
pT2	6	11
pT3	33	60
pT4	0	0
**Grade**		
G1	2	4
G2	32	58
G2-3	1	2
G3	18	33
G3-4	1	2
G4	1	2
**Surgical margins**		
R0/Rx	47	85
R1	8	15
**Venous invasion**		
V0/Vx	48	87
V1	5	9
V2	2	4
**Lymph invasion**		
L0/Lx	45	87
L1	7	13
**Lymph node metastasis**		
N0/Nx	51	98
N1	4	8
N2	0	0
**Metastasis**		
M0	47	85
M1	8	15

Supplementary Table 2: Clinical characteristics of ccRCC patients.

Characteristics	Microarray Patients (n=28)	%
**Age, years**		
Median	65	
Range	40-78	
**Sex**		
Male	19	70
Female	8	30
**Pathological stage**		
pT1	12	44
pT2	1	4
pT3	13	48
pT4	1	4
**Grade**		
G1	1	4
G2	19	70
G2-3	0	0
G3	7	26
G3-4	0	0
G4	0	0
**Surgical margins**		
R0/Rx	27	100
R1	0	0
**Venous invasion**		
V0/Vx	20	74
V1	6	22
V2	1	4
**Lymph invasion**		
L0/Lx	23	85
L1	4	15
**Lymph node metastasis**		
N0/Nx	25	92
N1	1	4
N2	1	4
**Metastasis**		
M0	14	52
M1	13	48

Supplementary Table 3: IC50-values of spheres treated with ICG-001 or DAPT.

Sphere culture	ICG-001 (µM)	DAPT (µM)
ccRCC54	5.96	9.34
ccRCC02	6.28	>100
ccRCC55	6.48	43.74
ccRCC52	6.54	>100
ccRCC45	6.77	7.67
ccRCC49	7.28	>100
ccRCC71	9.52	28.10
ccRCC42	9.71	9.51
ccRCC38	9.85	121.30
ccRCC39	10.41	57.56
ccRCC70	10.57	42.42
ccRCC37	11.09	21.97
ccRCC34	11.45	23.64
ccRCC32	12.68	88.19
ccRCC76	14.22	81.25
ccRCC24	15.94	38.67
ccRCC33	16.68	90.20
ccRCC77	19.23	>100
ccRCC60	19.97	4.43
ccRCC27	21.46	>100
ccRCC78	21.93	75.67
ccRCC21	22.94	>100
ccRCC16	23.75	9.59
ccRCC41	23.88	95.42
ccRCC12	24.98	66.86
ccRCC44	25.60	20.09
ccRCC50	30.00	69.24
ccRCC79	32.81	NA
ccRCC61	32.99	48.76
ccRCC68	35.33	>100
ccRCC25	35.88	>100
ccRCC04	>50	11.79
ccRCC30	>50	32.35
ccRCC29	>50	38.62
ccRCC62	>50	65.02
ccRCC26	>50	69.21
ccRCC65	>50	75.55
ccRCC73	>50	75.55
ccRCC57	>50	81.51
ccRCC36	>50	117.10
ccRCC75	>50	>100
ccRCC17	>50	NA
ccRCC74	NA	23.60
ccRCC03	NA	77.40
ccRCC46	NA	91.06
ccRCC18	NA	>100
ccRCC43	NA	>100
ccRCC56	NA	>100

Supplementary Table 4. Correlation of IC50 values with pathological stage, Fuhrman Grade or percentage of $CXCR4^{+}MET^{+}CD44^{+}$.

	Spearman correlation coefficient	p-value (Spearman correlation)
**Correlation with ICG-001 (n=44)**		
Pathological stage	0.04	0.83
Fuhrmann Grade	-0.19	0.25
% $CXCR4^{+}/MET^{+}/CD44^{+}$	0.15	0.54
**Correlation with DAPT (n=44)**		
Pathological stage	-0.14	0.36
Fuhrmann Grade	0.24	0.12
% $CXCR4^{+}/MET^{+}/CD44^{+}$	0.20	0.67

Supplementary Table 5: RT-qPCR primer sequences.

Target gene	Forward primer sequence	Reverse primer sequence
AXIN2	CTGGCTATGTCTTTGCACCA	CTTCACACTGCGATGCATTT
CD44	GGCTTTCAATAGCACCTTGC	ACACCCCTGTGTTGTTTGCT
CTNNB1	ATTCTTGGCTATTACGACAGACTG	TTACTAAAGCTTGGGGTCCACC
GAPDH	GAGTCAACGGATTTGGTCGT	TTGATTTTGGAGGGATCTCG
HES1	CGGACATTCTGGAAATGACA	CATTGATCTGGGTCATGCAG
HES2	GCATCAACCAGAGCCTGAG	TCCAGGACGTCTGCCTTCT
HEY1	TGGATCACCTGAAAATGCTG	ATGCGAAACCAGGTCGAACTC
HEY2	AAGATGCTTCAGGCAACAGG	TACCGCGCAACTTCTGTTAG
JAG1	GAACCCGATCAAGGAAATCA	GAGCTCAGCAAGGGAACAAG
LEF1	CAAGCACAAACCTCTCAGGA	TGGGTGGAGAAAGAGATCCA
LGR4	AACAGTACCCAGTGAAGCCATT	ACCTCCGTCAAGCTGTTGTC
NOTCH1	CACACCAACGTGGTCTTCAA	CGGTTGTCAATCTCCAGGTA
NOTCH3	AGGATGTGGACGAGTGCTCTAT	CAGATACAGGTGAACTGGCCTA
SOX9	CCAACGCCATCTTCAAGG	AAGTCGATAGGGGGCTGTCT
TCF7L2	TCTAACAAAGTGCCAGTGGTG	GGCGATAGTGGGTAATACGG

Supplementary Table 6: List of antibodies used for FACS, IF, IHC, and MACS

ID	Antibody	SOURCE	Application	Order-No. and RRID
1	Mouse anti-CD44 FITC	BD Biosciences	FACS	555478, RRID:AB_395870
2	Mouse anti-CXCR4	R&D	FACS	MAB170, RRID: AB_357856
3	Rabbit anti-c-Met	Santa Cruz	FACS	Sc-160, RRID: N/A
4	Mouse anti-CD105 PE	Biolegend	FACS	323206, RRID:AB_755958
5	Streptavidin-APC	Life Technologies	FACS	SA1005
6	Goat anti-mouse biotinylated	Sigma Aldrich	FACS	B7022, RRID: AB_258598
7	Anti-Rabbit Pacific Blue	Invitrogen	FACS	P-10994, RRID: AB_2539814
8	Mouse anti-CD24 FITC	BD Biosciences	FACS	560992, RRID: AB_10562033
9	Mouse anti-CD29 PE-Cy5	BD Biosciences	FACS	559882, RRID: AB_397356
10	Mouse anti-CD133 biotinylated	Miltenyi Biotec	FACS	130-098-897, RRID: AB_2660890
11	Mouse anti-CD90 APC	BD Biosciences	FACS	561971, RRID: Ab_10898350
12	Mouse anti-E-Cadherin	BD Biosciences	FACS	610181, RRID: AB_397580
13	Anti-Epcam	Abcam	FACS/IF	Ab223582, RRID: AB_2762366
14	Rabbit anti-Carbonic anhydrase IX	Abcam	IF	ab128883, RRID: N/A
15	Mouse anti-CD44	Cell Signaling Technology	IF	3570, RRID: AB_2076465
16	Rabbit anti-JAG1	Atlas Antibodies	IF	HPA021555, RRID: AB_1851969
17	Rabbit anti-Met	Cell Signaling Technology	IF	8198, RRID: AB_10858224
18	Mouse anti-CD10	Dako	IF	M7308, RRID: AB_2144424
19	Rabbit anti-VCAM1	Abcam	IF	ab134047, RRID:AB_2721053
20	Rabbit anti-Ki-67	Thermo Fisher	IF/IHC	RM-9106-S, RRID: AB_149792
21	Rabbit anti-NOTCH1	Rockland Inc.	IHC	100-401-407, RRID: AB_2153490
22	Mouse anti-beta-catenin	BD biosciences	IHC	610153, RRID: AB_397554
23	Mouse anti-Calbindin	Sigma Aldrich	IHC	C9848, RRID:AB_2314065
24	CD45 Microbeads	Miltenyi Biotec	MACS	130-045-801

FACS, Fluorescence-activated cell sorting, IF, immunofluorescence,
IHC, immunohistochemistry, MACS magnetic-activated cell sorting
