# Supplementary material for: Inhibiting WNT and NOTCH in renal cancer stem cells and the implications for human patients
Source: Nat Commun. 2020 Feb 17;11:929. doi: 10.1038/s41467-020-14700-7 (PMC7026425; doi:10.1038/s41467-020-14700-7)
Supplement: Supplementary file 3 — Description of Additional Supplementary Files [file 41467_2020_14700_MOESM3_ESM.pdf]

Description of Additional Supplementary Files

File Name: Supplementary Data 1
Description: Differentially regulated genes in CXCR4+MET+CD44+ cells and sphere cells. p-values were corrected for multiple testing using Benjamini and Hochberg method.
