# Supplementary material for: Inhibiting WNT and NOTCH in renal cancer stem cells and the implications for human patients
Source: Nat Commun. 2020 Feb 17;11:929. doi: 10.1038/s41467-020-14700-7 (PMC7026425; doi:10.1038/s41467-020-14700-7)
Supplement: Supplementary file 5 — Reporting Summary [file 41467_2020_14700_MOESM5_ESM.pdf]

nature research

Corresponding author(s): Walter Birchmeier

Last updated by author(s): 01/19/2020

Reporting Summary

Nature Research wishes to improve the reproducibility of the work that we publish. This form provides structure for consistency and transparency in reporting. For further information on Nature Research policies, see Authors & Referees and the Editorial Policy Checklist .

Statistics

For all statistical analyses, confirm that the following items are present in the figure legend, table legend, main text, or Methods section.

n/a	Confirmed	
☐	☒	The exact sample size (*n*) for each experimental group/condition, given as a discrete number and unit of measurement
☐	☒	A statement on whether measurements were taken from distinct samples or whether the same sample was measured repeatedly
☐	☒	The statistical test(s) used AND whether they are one- or two-sided<br>*Only common tests should be described solely by name; describe more complex techniques in the Methods section.*
☒	☐	A description of all covariates tested
☐	☒	A description of any assumptions or corrections, such as tests of normality and adjustment for multiple comparisons
☐	☒	A full description of the statistical parameters including central tendency (e.g. means) or other basic estimates (e.g. regression coefficient) AND variation (e.g. standard deviation) or associated estimates of uncertainty (e.g. confidence intervals)
☐	☒	For null hypothesis testing, the test statistic (e.g. *F*, *t*, *r*) with confidence intervals, effect sizes, degrees of freedom and *P* value noted<br>*Give P values as exact values whenever suitable.*
☒	☐	For Bayesian analysis, information on the choice of priors and Markov chain Monte Carlo settings
☒	☐	For hierarchical and complex designs, identification of the appropriate level for tests and full reporting of outcomes
☐	☒	Estimates of effect sizes (e.g. Cohen's *d*, Pearson's *r*), indicating how they were calculated

Our web collection on statistics for biologists contains articles on many of the points above.

Software and code

Policy information about availability of computer code

Data collection	
Data analysis	Single Sequencing raw BCL files were converted to FASTQ using the BCL2FASTQ software (Illumina). FASTQ files were processed using a multistep python library available on GitHub (https://github.com/yanailab/celseq2). Using BowTie2, reads were mapped to the Hg19 reference genome, and raw reads were counted using a modified HTSeq-count script. Data were then analyzed using the Seurat package in R (http://satijalab.org/seurat/).<br>HumanHT-12 v4 bead chip data were normalized and log-transformed in Partek Genomics Suite (Partek) and analyzed using the limma package87 in R.<br>GO term enrichment and Kegg pathway analyses were performed with DAVID (see references in text).<br>Expression of stem cell genes was analyzed using the GeneSpring GX software (Agilent), and supervised hierarchical clustering was performed in Genesis (see references in text).

For manuscripts utilizing custom algorithms or software that are central to the research but not yet described in published literature, software must be made available to editors/reviewers. We strongly encourage code deposition in a community repository (e.g. GitHub). See the Nature Research guidelines for submitting code & software for further information.

Data

Policy information about availability of data

All manuscripts must include a data availability statement. This statement should provide the following information, where applicable:

- Accession codes, unique identifiers, or web links for publicly available datasets
- A list of figures that have associated raw data
- A description of any restrictions on data availability

The Gene Expression omnibus accession number is GSE89461 for Illumina bead chip experiments, GSE66270 and GSE66271 for gene expression from fresh frozen ccRCC tissues and GSE110680 for single-cell sequencing. Data with associated raw data are shown in Figures 4 and 5, as well as Supplementary Figures 4 and 5. There are no restrictions on data availability.

Field-specific reporting

Please select the one below that is the best fit for your research. If you are not sure, read the appropriate sections before making your selection.

☒ Life sciences ☐ Behavioural & social sciences ☐ Ecological, evolutionary & environmental sciences

For a reference copy of the document with all sections, see nature.com/documents/nr-reporting-summary-flat.pdf

Life sciences study design

All studies must disclose on these points even when the disclosure is negative.

Sample size	For xenograft assays, we estimated that we would need at least three samples per treatment group to see a two-fold change in tumor volume, for a power of 80% and for the probability of type I error (α) = 0.05. Power calculations were performed in G*Power 3. No sample size calculations were performed for other experiments.
Data exclusions	Data were not excluded from analysis.
Replication	Microarray data from FAC-sorted and sphere cultures were validated in an independent set of patient-derived primary cells to confirm deregulated gene expression. Kaplan-Meier Analysis from a limited number of ccRCC patients was confirmed in the TCGA data set. For all other experiments, we performed experiments in multiple patient-derived primary cells to ensure reproducibility.
Randomization	For inhibitor assays in xenografts, subcutaneous tumors were allocated randomly to treatment and control groups.
Blinding	Blinding was not performed.

Reporting for specific materials, systems and methods

We require information from authors about some types of materials, experimental systems and methods used in many studies. Here, indicate whether each material, system or method listed is relevant to your study. If you are not sure if a list item applies to your research, read the appropriate section before selecting a response.

Materials & experimental systems

n/a	Involved in the study	
☐	☒	Antibodies
☐	☒	Eukaryotic cell lines
☒	☐	Palaeontology
☐	☒	Animals and other organisms
☐	☒	Human research participants
☐	☒	Clinical data

Methods

n/a	Involved in the study	
☒	☐	ChIP-seq
☐	☒	Flow cytometry
☒	☐	MRI-based neuroimaging

Antibodies

Antibodies used	See attached list
Validation	Antibodies were selected based on previous publications in ccRCC whenever possible. When antibodies were used for staining of organoids or spheres there specificity was validated on tissue section to account for correct cellular localization. Antibodies for FACS were titrated and IgG controls were used.

Eukaryotic cell lines

Policy information about cell lines

Cell line source(s)	Only primary patient-derived cells were used in this study
Authentication	Primary patient-derived cells were not authenticated.
Mycoplasma contamination	Primary patient-derived cells were not tested for mycoplasma contamination.
Commonly misidentified lines (See ICLAC register)	*Name any commonly misidentified cell lines used in the study and provide a rationale for their use.*

Animals and other organisms

Policy information about studies involving animals; ARRIVE guidelines recommended for reporting animal research

Laboratory animals	Subcutaneous patient-derived xenografts were established from 4x4 mm ccRCC tissue cubes in adult nude (Nu/J) mice and all subsequent experiments were performed in adult NSG (NOD.Cg-Prkdcscid Il2rgtm1Wjl/SzJ, NOD scid gamma) mice. Both sexes were used.
Wild animals	*Provide details on animals observed in or captured in the field; report species, sex and age where possible. Describe how animals were caught and transported and what happened to captive animals after the study (if killed, explain why and describe method; if released, say where and when) OR state that the study did not involve wild animals.*
Field-collected samples	*For laboratory work with field-collected samples, describe all relevant parameters such as housing, maintenance, temperature, photoperiod and end-of-experiment protocol OR state that the study did not involve samples collected from the field.*
Ethics oversight	Animal experiments were carried out in accordance with the of the German Animal Protection Law and approved by the local responsible authorities. Epo complies to the EU guideline “European convention for the protection of vertebrate animals used for experimental and other scientific purposes. (EST 123)”. Further we handle our animals according to the “Regulation on the protection of experimental scientific purposes or other Purposes used animals”. Compliance with the above rules and regulations is monitored by the Landesamt fuer Gesundheit und Soziales (LAGeSo) which is the responsible regulatory authority monitoring the animal husbandry based on the German Animal Welfare Act. Approval was given after careful inspection of the site including bedding, feeding & water, ventilation, temperature & humidity, cleaning and hygiene concepts.

Note that full information on the approval of the study protocol must also be provided in the manuscript.

Human research participants

Policy information about studies involving human research participants

Population characteristics	All patient characteristics are summarized in Supplementary Table 1 and 3.
Recruitment	All patients undergoing nephrectomy at the Charite - University Hospital from 2011 to 2018 were included if tumor size and localization allowed for the collection of tissue. Participants were exluded if they had a known subtype other than ccRCC or if a subtype other than ccRCC was diagnosed post surgery.
Ethics oversight	The project was approved by the ethics committee of the Charite - University Hospital (EA1/134/12) and informed consent was obtained from all patients.

Note that full information on the approval of the study protocol must also be provided in the manuscript.

Clinical data

Policy information about clinical studies

All manuscripts should comply with the ICMJEguidelines for publication of clinical research and a completedCONSORT checklist must be included with all submissions.

Clinical trial registration	*Provide the trial registration number from ClinicalTrials.gov or an equivalent agency.*
Study protocol	*Note where the full trial protocol can be accessed OR if not available, explain why.*
Data collection	*Describe the settings and locales of data collection, noting the time periods of recruitment and data collection.*
Outcomes	*Describe how you pre-defined primary and secondary outcome measures and how you assessed these measures.*

Flow Cytometry

Plots

Confirm that:

- [ ] The axis labels state the marker and fluorochrome used (e.g. CD4-FITC).
- [ ] The axis scales are clearly visible. Include numbers along axes only for bottom left plot of group (a 'group' is an analysis of identical markers).
- [x] All plots are contour plots with outliers or pseudocolor plots.
- [x] A numerical value for number of cells or percentage (with statistics) is provided.

Methodology

Sample preparation	ccRCC tissue samples were processed within 24 hrs after surgery. Briefly, the tissue was extensively washed in PBS, minced into cubes smaller than 1 mm3, digested enzymatically using collagenase P and filtered through sieves with 100 and 40 µm pore size. Subsequently, erythrocytes were lysed and leukocytes were depleted by MACS using anti-CD45 micro-beads (Milteniy BioTec).
Instrument	Isolated tumors cells were analyzed with FACS Aria I or Aria F Cell Sorters (BD Biosciences, Germany).
Software	Data were analyzed using Flowjo
Cell population abundance	Purity of populations was confirmed by reanalyzing a fraction of FAC-sorted cells. Purity was above 95% for all experiments (resorted cells within the gates).
Gating strategy	Single cells were gated in FSC/SSC plots and singlets were confirmed by plotting FSC-H vs. FSC-A and SSC-H vs SSC-A. Viable cells were identified by 7-AAD staining. For identifcation of triple positive cells, CXCR4+MET+ cells were gated and then analyzed for CD44. Gates were defined by position of cells stained with suitable IgG control (negativ control).

- [x] Tick this box to confirm that a figure exemplifying the gating strategy is provided in the Supplementary Information.
